# Assessment of the Diagnostic Reliability of Modified Alvarado Scores and Abdominal Ultrasonography in Acute Appendicitis

**DOI:** 10.7759/cureus.38991

**Published:** 2023-05-14

**Authors:** Piyush Bhardwaj, Aparna Behura, Ipsa Mohapatra, Chinmaya Behera, Subrat Mohanty, Amaresh Mishra, Bandita Panda, Narella S Krishna, K Ranjit

**Affiliations:** 1 General Surgery, Radha Devi Jageshwari Memorial Medical College and Hospital, Muzaffarpur, IND; 2 Pathology, Kalinga Institute of Medical Sciences, PBM Hospital, Bhubaneswar, IND; 3 Community Medicine, Kalinga Institute of Medical Sciences, Bhubaneswar, IND; 4 Surgery, Kalinga Institute of Medical Sciences, Bhubaneswar, IND; 5 Pediatric Surgery, Kalinga Institute of Medical Sciences, Bhubaneswar, IND; 6 Research and Development, Kalinga Institute of Medical Sciences, Bhubaneswar, IND; 7 General Surgery, Kalinga Institute of Medical Sciences, PBM Hospital, Bhubaneswar, IND

**Keywords:** diagnostic reliability, diagnosis, ultrasonography, alvarado score, acute appendicitis

## Abstract

Introduction

Acute appendicitis is a common surgical emergency. Clinical assessment plays a major role; however, subtle clinical features in early stages and atypical presentation makes diagnosis challenging. Ultrasonography (USG) of the abdomen is a usual investigation that aids in diagnosis, however, it is operator dependent. A contrast-enhanced computed tomography (CECT) of the abdomen is more accurate; however, it exposes the patient to hazardous radiation. The study aimed to combine clinical assessment and USG abdomen in the reliable diagnosis of acute appendicitis.

Objectives

The objective of this study was to assess the diagnostic reliability of the Modified Alvarado Score and ultrasonography of the abdomen in acute appendicitis.

Material and methods

All patients with right iliac fossa pain, clinically suspected of having acute appendicitis, admitted to the department of general surgery, Kalinga Institute of Medical Sciences (KIMS), Bhubaneswar, between January 2019 and July 2020, who gave consent were included. Clinically, Modified Alvarado Score (MAS) was calculated, after which patients were subjected to USG abdomen, where findings were noted and a sonologic score was calculated. The study group was the patients who needed appendicectomy (n=138). Operative findings were noted. Histopathological diagnosis of acute appendicitis was deemed as confirmatory in these cases and was correlated with MAS and USG scores to determine diagnostic accuracy.

Results

A combined clinicoradiological (MAS + USG) score of seven showed a sensitivity of 81.8% and a specificity of 100%. The specificity of score seven or above was 100%; however, the sensitivity at 81.8%. The diagnostic accuracy of the clinicoradiological was 87.5%. The negative appendicectomy rate was 4.34%, with a diagnosis of acute appendicitis being confirmed for 95.7% of patients upon histopathological examination.

Conclusion

The MAS and USG of the abdomen, which is an affordable and non-invasive tool, showed increased diagnostic reliability, and hence it can help reduce the use of CECT abdomen, as CECT abdomen is considered as a gold standard for confirmation or exclusion of diagnosis of acute appendicitis. Use of the combined scoring system of MAS and USG abdomen can be used as a cost-effective alternative.

## Introduction

Acute appendicitis, which has a lifetime prevalence of approximately one in seven worldwide, presents as an abdominal surgical emergency in which a delay or missed diagnosis can lead to complications resulting in morbidity and mortality [1]. With clinical assessment playing a major role in diagnosis and treatment, subtle clinical features in early stages and atypical presentation make diagnosis challenging, even for an experienced clinician [2]. Tests and procedures used to diagnose appendicitis include a wide range of options from physical examination, blood tests, and urine tests to imaging tests such as an abdominal X-ray, abdominal ultrasound, computerized tomography scan, or magnetic resonance imaging to help confirm appendicitis or find other causes for the patient's pain [3]. With the limitations of either a single test or a combination of tests to distinguish all cases of acute appendicitis from other conditions, an awareness of the limitations of imaging, blood tests, and scoring systems is therefore essential because diagnosis is still a challenge.

While some studies have shown that the Modified Alvarado Scoring (MAS) system provided a higher degree of diagnostic accuracy in patients suspected of acute appendicitis and reduced negative appendicectomy and complication rates [4-6], another study showed that higher scores performed poorly in predicting the diagnosis of acute appendicitis preoperatively and in reducing negative appendicectomies [7]. Kalan et al. found that a high score was an easy and satisfactory aid to early diagnosis of appendicitis in children and men but produced a false-positive rate in women [8]. A meta-analysis of 18 studies showed that an abdominal ultrasound (USG) has significant accuracy of diagnosis in patients with suspected acute appendicitis [9]. The MAS comprises symptoms (migration of pain, anorexia, and nausea), physical signs (right lower quadrant tenderness, rebound tenderness, and pyrexia), and laboratory values (leucocytosis) with a total score of nine. A score of seven or more is considered highly suggestive of acute appendicitis. USG scoring criteria are based on reports by Stephens et al., Harrison et al., Fu et al. [1, 2, 9]; a sonological score of more than six is diagnostic of appendicitis. The present study was planned to determine the reliability of already existing MAS and USG findings individually and together in the diagnosis of acute appendicitis correlating with the operative findings and histopathological outcome of the appendix specimen.

Objectives

The objectives of this study were to assess the diagnostic reliability of the Modified Alvarado Score and ultrasonography of the abdomen in acute appendicitis.

## Materials and methods

A longitudinal study was undertaken in the department of general surgery, Kalinga Institute of Medical Sciences (KIMS), Kalinga Institute of Industrial Technology (KIIT) University, Bhubaneswar. The study period was from January 2019 to July 2020.

The study population comprised all patients admitted with right iliac fossa (RIF) pain, suspected of having acute appendicitis and needing appendicectomy. Suspicion of acute appendicitis was based on the symptoms of pain in RIF, nausea, and/or vomiting with a positive Mcburney's point tenderness (elicited by the surgeon).

Inclusion and exclusion criteria

All cases of acute appendicitis, adult and pediatric groups were included in the study - ages 5 to 80 years, of both sexes, undergoing appendicectomy in the department of general surgery KIMS, Bhubaneswar. Patients who had an appendicular phlegmon, appendicular abscess, recurrent appendicitis, or cases of interval appendicectomy were excluded. Patients with pain in the right lower abdomen with demonstrable pathology other than acute appendicitis (these were patients who initially presented with pain right lower abdomen, later on diagnosed to have non-appendicular pathology), and those who had a previous history of appendicitis were also excluded from the study.

**Figure 1:**
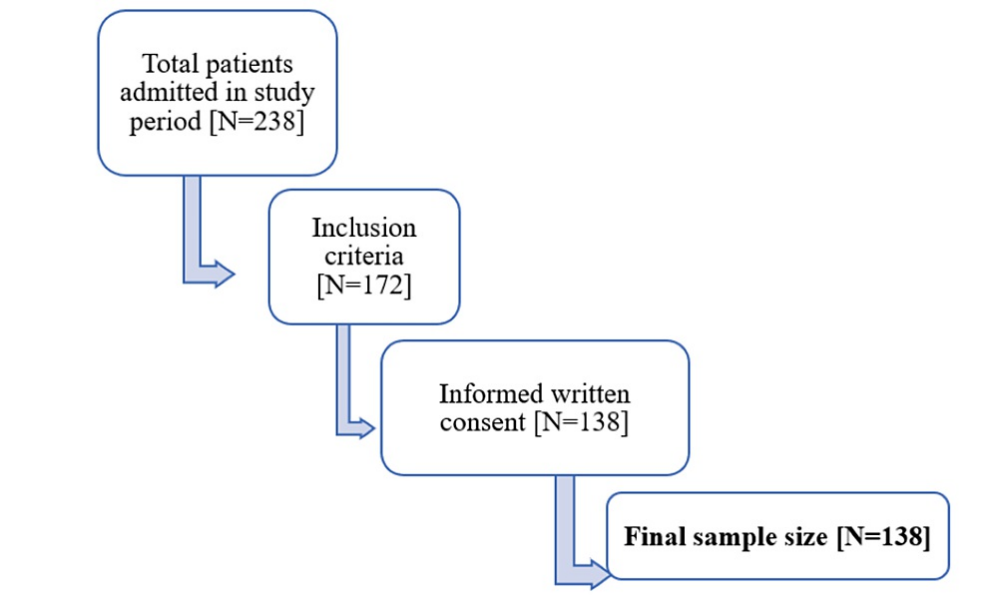
Sample population included in the study

The sample size comprised all patients satisfying the inclusion criteria and providing informed written consent during the data collection period of January 2019 to July 2020 (Figure 1)

A convenience sampling technique was used. For all patients reporting with RIF pain and suspected of having acute appendicitis who were admitted to the department of general surgery, Kalinga Institute of Medical Sciences (KIMS), a MAS was calculated (as per the conventional scoring, i.e., symptoms (migration of pain (score 1), anorexia (score 1) and nausea (score 1)), physical signs (right lower quadrant tenderness (score 2), rebound tenderness (score 1), and pyrexia (score 1)), and laboratory values (leucocytosis (score 2)) for a total score of nine. A score of seven or more is considered highly suggestive of acute appendicitis. Similarly, patients were subjected to USG abdomen, findings were noted, and appropriate sonologic scores were calculated (anteroposterior diameter > 6mm (score 1), non-compressible tubular structure (score 1), echogenic changes in the periappendicular fat (score 1), thickness of appendicular wall > 2mm (score 1), target sign (score 1), appendicolith (score 1) for a total score of six [10]. Histopathological diagnosis of acute appendicitis was regarded as confirmatory in these cases, and it was correlated with individual MAS and USG scoring, and the two combined to arrive at a diagnosis. Diagnostic measures such as sensitivity, specificity, area under the receiver operating characteristic (ROC) curve, and appropriate cut-off for MAS and USG scores individually and combined were reported.

The study tool used was a researcher-made format to collect information regarding patient details (name, age, sex, address, occupation), duration of the symptoms, associated comorbid conditions, history of previous episodes of abdomen pain, history of any past abdominal surgery, physical examination (general examination, icterus, pallor, temperature, pulse, BP, etc.), local examination (detailed per-abdominal examination findings were noted), laboratory parameters (complete blood count, blood sugar, viral markers, liver function test, renal function test, coagulation profile), and imaging studies (USG abdomen findings such as the antero-posterior diameter of the appendix, echogenic changes in the peri-appendicular fat, thickness of the appendicular wall, tubular structure whether non-compressible and presence of appendicolith, target sign, etc). We calculated MAS and USG scores for each patient.

Ethical implications

The study was presented before the institutional research and ethics committee, and approval was obtained (KIMS/KIIT/IEC/035/2018). All the procedures followed were in accordance with the ethical standards of the Institutional Ethics Committee and with the Declaration of Helsinki 1975

Data analysis

We entered all collected data into a Microsoft Excel (Microsoft, Redmond, Washington) spreadsheet and analyzed it using EPI Info statistical software (version 7.3.2). We drew the ROC curve using an Excel sheet and reported diagnostic measures such as sensitivity, specificity, area under the ROC curve, and the appropriate cut-off for MAS and USG scores individually and combined. We calculated sensitivity and specificity for both scores with histopathologically determined appendicitis. We calculated the ROC curves to represent the ratio of true versus false positives individually for MAS and USG scores and for both together.

## Results

Of the 138 patients admitted through the general emergency or the outpatient department of general surgery with a clinical diagnosis of acute appendicitis who satisfied the inclusion criteria and provided consent, 73% were males, and 33.33% were in the adolescent age group of 11 to 20 years; 46.74% had a USG score of >2 (Table 1).

**Table 1:** Demographic and diagnostic features of the participants (N=138) *In 46 of the patients, the clinical findings were deemed confirmatory and a decision to proceed with urgent appendicectomy was made without radiological investigation. For the rest of the patients (n=92), clinical examination and ultrasound abdomen were both conducted USG - ultrasonography

Variables	Frequency in number	Frequency in percentage
Age group (in years)		
5-10	7	5.07
11-20	46	33.33
21-30	36	26.09
31-40	23	16.67
41-50	10	7.25
51-60	11	7.97
>61	5	3.62
Gender		
Male	101	73.19
Female	37	26.81
USG score (N=92)*		
0	4	4.35
1	16	17.39
2	43	46.74
3	27	29.35
4	2	2.17
Modified Alvarado Score		
2	1	0.72
3	9	6.52
4	19	13.77
5	29	21.01
6	45	32.62
7	34	24.64
8	1	0.72
Modified Alvarado Score + USG Score (N=92)*		
4	4	4.35
5	6	6.50
6	10	10.87
7	17	18.48
8	24	26.10
9	23	25.00
10	8	8.70

**Figure 2:**
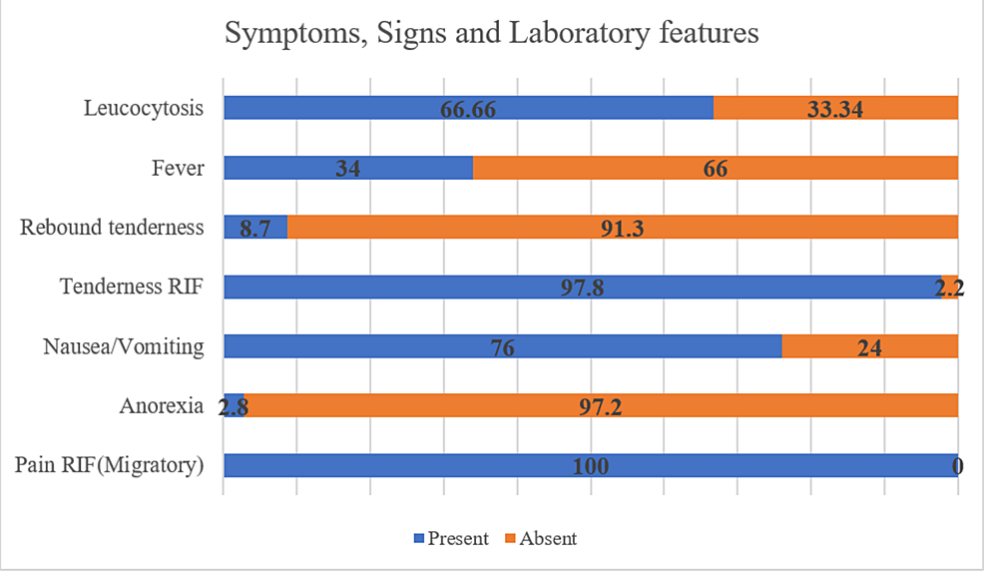
Presenting symptoms and signs at the time of admission (N=138) RIF - right iliac fossa

Pain in the RIF was the most common symptom and present in all followed by the clinical sign of tenderness in the RIF (97.8%; Figure 2)

In 70.29% of the cases, the position of the appendix was retrocaecal; in 62.31%, it was inflamed and oedematous. 94.93% of the patients had a histopathological finding of acute appendicitis (Table 2).

**Table 2:** Intraoperative and histopathological findings regarding the participants (N=138)

Variables	Frequency in number	Frequency in percentage
Position of appendix		
Retrocaecal	97	70.29
Pelvic	28	20.29
Subcaecal	6	4.35
Pre-ileal	5	3.62
Post-ileal	2	1.45
Intraoperative findings		
Inflamed and oedematous appendix	86	62.31
Appendicular perforation	26	18.84
Faecolith	7	5.00
Gangrenous appendix	7	5.00
Mucocele	2	1.40
Normal appendix		
Histopathological findings		
Acute appendicitis	131	94.93
Normal appendix	3	2.17
Carcinoid tumour	2	1.46
Mucinous cystadenoma	1	0.72
Granulomatous appendicitis	1	0.72

**Figure 3:**
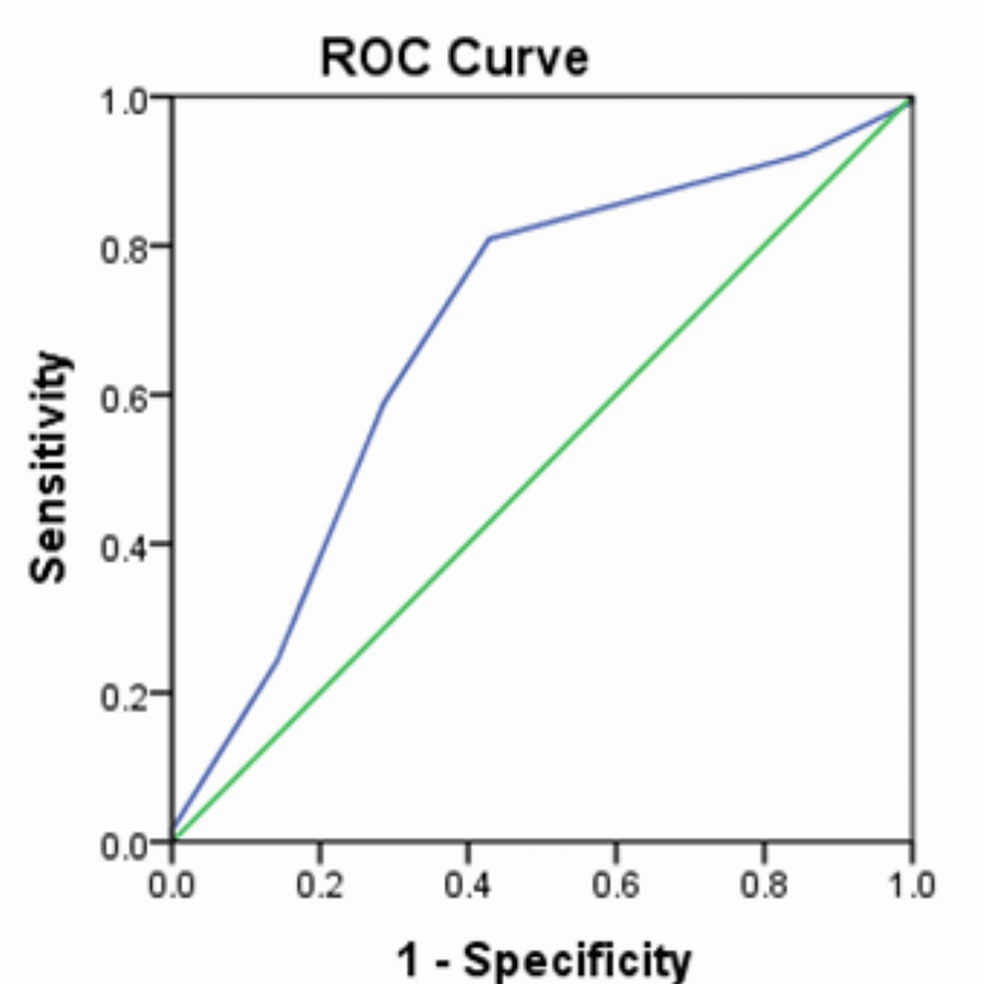
Receiver operating characteristic curve showing the diagnostic accuracy of the Modified Alvarado Score Area under curve = 0.686; (0.468-0.903 95% confidence interval)

The diagnostic accuracy of MAS was 68.6% (Figure 3). At a MAS of four, sensitivity was 80.9% and specificity 57.1%. At a score of five, sensitivity was 58.8% and specificity 71.4% (Table 3).

**Table 3:** Sensitivity and specificity of the Modified Alvarado Score

Modified Alvarado Score	Sensitivity (%)	Specificity (%)
1.00	1.000	0
2.00	99.2	0
3.00	92.4	14.3
4.00	80.9	57.1
5.00	58.8	71.4
6.00	24.4	85.7
7.00	1.5	100
8.00	0	100
9.00	0	100

**Figure 4:**
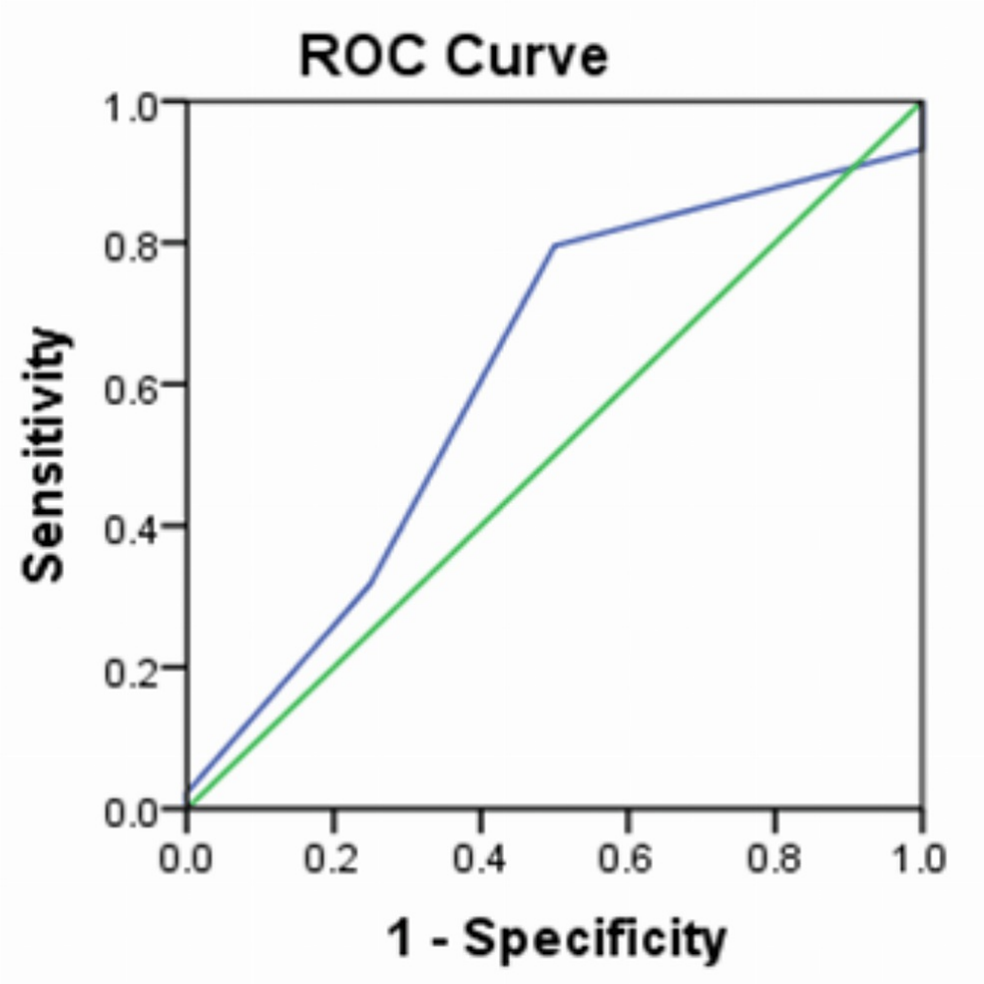
Receiver operating characteristic curve showing the diagnostic accuracy of the ultrasound score Area under curve = 0.614;(0.315- 0.912 95% confidence interval)

The diagnostic accuracy of the USG score was 61.6% (Figure 4). The sensitivity of the USG at a score of two was 79.5%, and the specificity was 50%. At a score of three, the sensitivity was 31.8% and the specificity 75% (Table 4).

**Table 4:** Sensitivity and specificity of the ultrasonography score

Ultrasonography score	Sensitivity	Specificity
0	1	0
1	93.2	0
2	79.5	50
3	31.8	75
4	23	100
5	0	100

**Figure 5:**
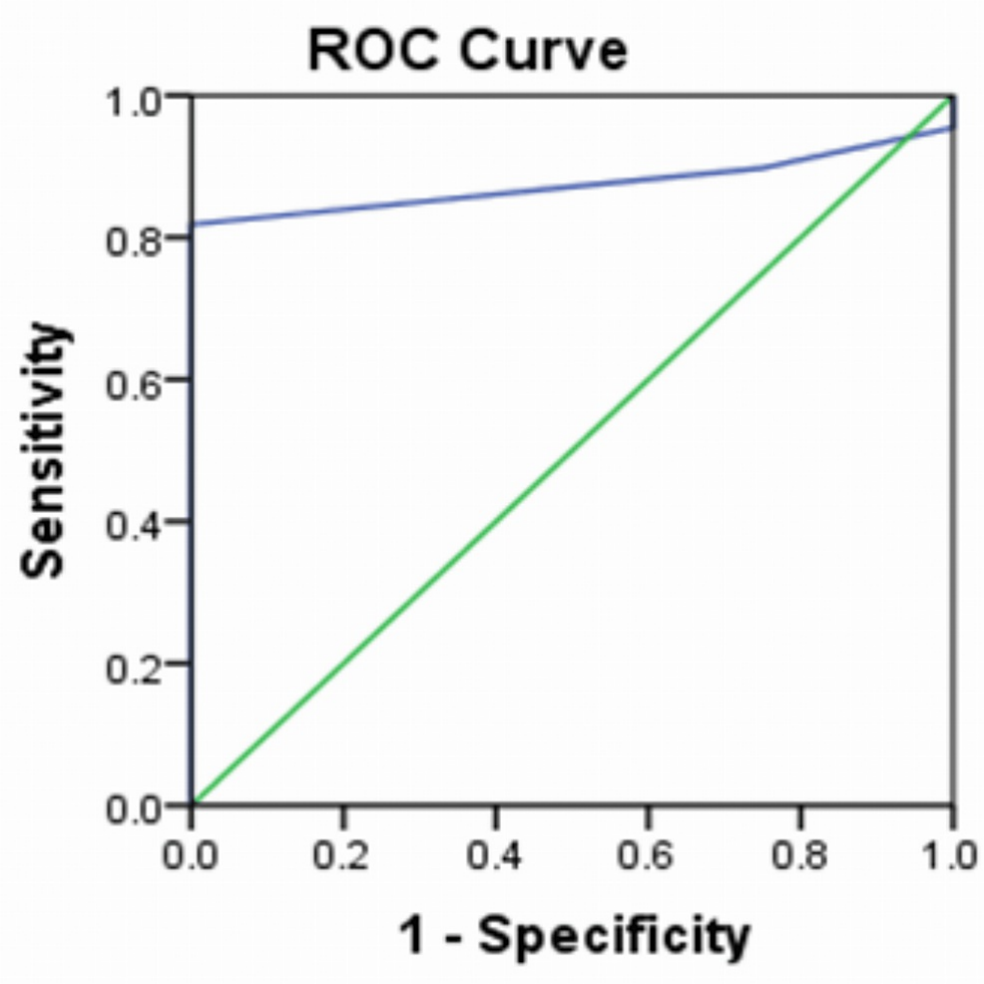
Receiver operating characteristic curve showing the diagnostic accuracy of the Modified Alvarado Score and ultrasonography score Area under curve = 0.875; (0.802-0.948 95% confidence interval)

The diagnostic accuracy of the MAS and USG scores was 87.5% (Figure 5).

The specificity at a combined clinicoradiological score of seven and above was consistently 100%. However, the sensitivity declined steadily from 81.8% at a score of seven to 9.1% at a score of 10 (Table 5). The MAS (with percentage of participants) were two (0.07%), three (6.5%), four (13.7%), five (21%), six (32.6%), seven (24.6%), and eight (0.07%). The histopathological findings were deemed confirmatory. The finding of appendicitis (n=93) and appendicitis along with periappendicitis (n=29) was found in an overwhelming majority of cases (n=123, 90%). A normal appendix was found in three cases (2.1%). There were two cases of carcinoid tumor (1.4%), a single case of mucinous cystadenoma (.7%), and one case of granulomatous appendicitis (0.7%). Based on the histopathology findings, the negative appendicectomy rate for our study was 4.34%.

**Table 5:** Sensitivity and specificity of the Modified Alvarado Score and ultrasonography score MAS - Modified Alvarado Score, USG - ultrasonography

MAS+USG score	Sensitivity	Specificity
4	100	0
5	95.5	0
6	89.8	25
7	81.8	100
8	62.5	100
9	35.2	100
10	9.1	100

In this study, the positive predictive value (PPV) for a combined clinicoradiological score of seven and above was 100%. At scores less than seven, the PPV was 83%. The overall PPV of the MAS and USG (abdomen) was 95.7%.

## Discussion

In this study, among 138 patients admitted with a diagnosis of acute appendicitis, 73% were male. The male predominance agrees with other studies [5-6]. However, in another study by Kanumba et al. conducted in Tanzania, there were more females (70.9%) [4]. These differences could be due to the regional variation in health-seeking behavior. In the current study, 33.33% of patients were in the adolescent age group of 11 to 20 years. This is similar to the findings of other studies [5, 6, 11]. It is well known that appendicitis is one of the most common presentations in cases of acute abdomen in this age group.

In the current study, the most common symptom was pain in the RIF, which was present in all patients, followed by the clinical sign of tenderness in the RIF (97.8%). In 70.29% of the cases, the position of the appendix was retrocaecal; in 62.31%, it was inflamed and oedematous. 94.93% of the patients had a histopathological finding of acute appendicitis with a negative appendicectomy rate of 4.34%. In a study by Kanumba et al., 66.9% had a positive histopathological finding and a higher negative appendicectomy rate of 33.1% [4]. These differences may be due to differences in the doctors' clinical acumen. While the former study was done in a tertiary care hospital, the latter was conducted in a medical setup wherein the authors cited misdiagnosis as the reason for the negative appendectomy rates. Another study by Nasiri et al. reported a similar histopathological finding of 89.3% [12].

In the present study, the diagnostic accuracy of MAS was 68.6%; at a MAS of four, sensitivity was 80.9% and specificity 57.1%. At a score of five, sensitivity was 58.8% and specificity 71.4%. A score of seven or more had 100% specificity. In a study done by Nasiri et al., the diagnostic accuracy was 62.6%, and at a score of seven, and the specificity was only 37.5% [12]. In a study by Kanaskar et al,, sensitivity was 41.94% and specificity 100%, at a score of seven [13]. In another study by Sridhar et al., the accuracy of the MAS was still lower at 47% [11]. Because the MAS is dependent on the doctor's clinical judgment, the differences may be ascertained to it.

In the current study, the diagnostic accuracy of the USG score was 61.6%. The sensitivity of the USG at a score of two was 79.5%, and the specificity was 50%. At a score of three, sensitivity was 31.8% and specificity 75%. In a study by Sridhar et al., the accuracy of the USG score was similar at 65% [11]. Nasiri et al. found a higher diagnostic accuracy at 72.4% [12]. In a study by Kanaskar et al., sensitivity was 74.19% [13]. Another study by Mishra et al. reported a sensitivity of 71.26 % [14]. Because USG is operator dependent, the results are prone to variability.

In the present study, the diagnostic accuracy of the MAS and USG score was 87.5%. The specificity at a combined clinicoradiological score of seven and above was consistently 100%. However, the sensitivity declined steadily from 81.8% at a score of seven to 9.1% at a score of 10. The PPV for combined clinicoradiological scores of seven and above was 100% in the study; at scores less than seven, it was 83%. Overall, the PPV of the MAS and USG was 95.7%. In a study by Kanaskar et al., the combined use of MAS and USG reported a sensitivity of 80.64% [13]. The current study findings corroborate the findings of other studies [15-17], adding to the evidence of the practice of using MAS and USG of the abdomen in diagnosing acute appendicitis.

## Conclusions

The diagnostic accuracy of MAS and USG score was 87.5 with a specificity of 100% at a combined clinicoradiological score of seven. The negative appendicectomy rate for MAS and USG (abdomen) in the study was 4.34%. The PPV for the combined clinicoradiological score of 7 and above was 100% in the study. At scores less than 7, it was 83%. Overall, the PPV of the MAS and USG (abdomen) was 95.7%. Hence, the use of contrast-enhanced computed tomography (CECT) abdomen can be reserved for cases where there is no significant ultrasonological findings, and the MAS is less than five. The judicious use of clinical scoring systems and USG of the abdomen, which is an affordable and non-invasive tool, can increase diagnostic reliability and reduce the use of CECT abdomen.
